# Combined Treatment with KV Channel Inhibitor 4-Aminopyridine and either γ-Cystathionine Lyase Inhibitor β-Cyanoalanine or Epinephrine Restores Blood Pressure, and Improves Survival in the Wistar Rat Model of Anaphylactic Shock

**DOI:** 10.3390/biology11101455

**Published:** 2022-10-03

**Authors:** Abdelouahab Bellou, Nacira Sennoun, Elhadi H. Aburawi, Richard L. Jayaraj, Seth L. Alper, Ibrahim Abdallah Alfaki, Javed Yasin, Subramanian Sekar, Mohamed Shafiuallah, Suhail Al-Salam, Abderrahim Nemmar, Elsadig Kazzam, Paul Michel Mertes, Suleiman Al-Hammadi

**Affiliations:** 1 Institute of Sciences in Emergency Medicine, Department of Emergency Medicine, Guangdong Provincial People’s Hospital, Guangdong Academy of Medical Sciences, Guangzhou 510080, China; 2 Department of Emergency Medicine, Wayne State University School of Medicine, Detroit, MI 48201, USA; 3 Department of Pediatrics, College of Medicine and Health Sciences, UAE University, Al-Ain P.O. Box 15551, United Arab Emirates; 4 Department of Cell and Molecular Biology, Team Cytokines and Nitric Oxide Synthases, Faculty of Biology, University HouariBoumediene USTHB, Bab-Ezzouar, Algiers 1611, Algeria; 5 Division of Nephrology and Vascular Biology Research Center, Beth Israel Deaconess Medical Center and Department of Medicine, Harvard Medical School, Boston, MA 02115, USA; 6 Department of Analytics in the Digital Era, UAE University, Al-Ain P.O. Box 15551, United Arab Emirates; 7 Department of Internal Medicine, College of Medicine and Health Sciences, UAE University, Al-Ain P.O. Box 15551, United Arab Emirates; 8 Renaissance LLC, Lakewood, NJ 08701, USA; 9 Department of Physiology, College of Medicine and Health Sciences, UAE University, Al-Ain P.O. Box 15551, United Arab Emirates; 10 Department of Pathology, College of Medicine and Health Sciences, UAE University, Al-Ain P.O. Box 15551, United Arab Emirates; 11 Pôle Anesthésie, Réanimation Chirurgicale, Hôpitaux Universitaires de Strasbourg, 67200 Strasbourg, France; 12 Faculté de Médecine de Strasbourg, FMTS (Fédération de Médecine Translationnelle de Strasbourg), Institut de Physiologie, Université de Strasbourg, UR 3072, 67200 Strasbourg, France; 13 College of Medicine, Mohammed Bin Rashid University of Medicine and Health Sciences (MBRU), Dubai Healthcare City, Dubai P.O. Box 505055, United Arab Emirates

**Keywords:** anaphylactic shock, Voltage-dependent K+ channel, 4-aminopyridine, β-cyanoalanine, dl-propargylglycine, angiotensin II, vasopressin, prostaglandins, leukotrienes

## Abstract

**Simple Summary:**

Allergic diseases are presenting a constant increase all over the world and caused by such different substances as food, drugs, and pollens. Anaphylactic shock is the more severe complication of allergy which can induce death if the treatment is not administered immediately. Some patients do not respond to the recommended treatment, intra venous or intramuscular epinephrine. The pathophysiology of anaphylactic shock is still under investigation. The mediators released after the activation of mast cells and basophiles act on endothelial cells and smooth muscle cells, inducing the vasodilation responsible for hypotension and shock. Nitric oxide and hydrogen sulphide are both intracellular mediators that induce vasodilation. The role of potassium voltage dependent channels is suspected. We aimed to demonstrate the ability of a blocker of potassium voltage dependent channels, 4-aminopyridine, alone or in combination with inhibitors of cystathionine γ-lyase to restore blood pressure and improve survival in an ovalbumin rat anaphylactic shock model. The blockade of potassium voltage dependent channels alone or combined with inhibitors of cystathionine γ-lyase, dl-propargylglycine, or β-cyanoalanine restored blood pressure and improved survival. These findings suggest possible investigative treatment pathways for research concerning epinephrine-refractory anaphylactic shock in patients.

**Abstract:**

The mechanism of anaphylactic shock (AS) remains incompletely understood. The potassium channel blocker 4-aminopyridine (4-AP), the inhibitors of cystathionine γ-lyase (ICSE), dl-propargylglycine (DPG) or β-cyanoalanine (BCA), and the nitric oxide (NO) synthase produce vasoconstriction and could be an alternative for the treatment of AS. The aim of this study was to demonstrate the ability of L-NAME, ICSE alone or in combination with 4-AP to restore blood pressure (BP) and improve survival in ovalbumin (OVA) rats AS. Experimental groups included non-sensitized Wistar rats (*n* = 6); AS (*n* = 6); AS (*n* = 10 per group) treated i.v. with 4-AP (AS+4-AP), epinephrine (AS+EPI), AS+DPG, AS+BCA, or with L-NAME (AS+L-NAME); or AS treated with drug combinations 4-AP+DPG, 4-AP+BCA, 4-AP+L-NAME, or 4-AP+EPI. AS was induced by i.v. OVA (1 mg). Treatments were administered i.v. one minute after AS induction. Mean arterial BP (MAP), heart rate (HR), and survival were monitored for 60 min. Plasma levels of histamine, prostaglandin E2 (PGE2) and F2 (PGF2α), leukotriene B4 and C4, angiotensin II, vasopressin, oxidative stress markers, pH, HCO3, PaO2, PaCO2, and K+ were measured. OVA induced severe hypotension and all AS rats died. Moreover, 4-AP, 4-AP+EPI, or 4-AP+BCA normalized both MAP and HR and increased survival. All sensitized rats treated with 4-AP alone or with 4-AP+BCA survived. The time-integrated MAP “area under the curve” was significantly higher after combined 4-AP treatment with ICSE. Metabolic acidosis was not rescued and NO, ICSE, and Kv inhibitors differentially alter oxidative stress and plasma levels of anaphylactic mediators. The AS-induced reduction of serum angiotensin II levels was prevented by 4-AP treatment alone or in combination with other drugs. Further, 4-AP treatment combined with EPI or with BCA also increased serum PGF2α, whereas only the 4-AP+EPI combination increased serum LTB4. Serum vasopressin and angiotensin II levels were increased by 4-AP treatment alone or in combination with other drugs. Moreover, 4-AP alone and in combination with inhibition of cystathionine γ-lyase or EPI normalizes BP, increases serum vasoconstrictor levels, and improves survival in the Wistar rat model of AS. These findings suggest possible investigative treatment pathways for research into epinephrine-refractory anaphylactic shock in patients.

## 1. Introduction

Allergic diseases are caused by a dysregulation of the immune system response to allergens [1]. Allergy became an important challenge for healthcare system in Western countries. Food allergy is increasing in the US with a prevalence of 10.8% [2]. The incidence of suspected perioperative anaphylaxis varies from to country to another country from one in 353 to one in 18,600 [3,4,5,6,7,8,9,10,11,12,13,14,15].

This incidence estimation is probably underestimated. It was suggested that the real incidence could be 1/7000 [16]. The perioperative anaphylaxis varies from 0 to 26.6% [6,8,17,18,19]. Delayed treatment with epinephrine is estimated at one-third anaphylaxis cases in Denmark [20]. In some cases, AS is refractory to epinephrin when a double or more than a triple dose of epinephrin is required and alternative drugs are needed as vasopressin, norepinephrine, metaraminol, or phenylephrine [21].

Cardiovascular dysfunction dominates the clinical presentation of systemic anaphylaxis, with hypotension and shock, arrhythmias, myocardial dysfunction, and cardiac arrest, which may lead to death if resuscitation is delayed [22,23,24,25,26,27,28].

Anaphylactic reactions are mediated by the allergen interaction of immunoglobulin E (IgE) pre-bound with the FcεRI high-affinity IgE receptor expressed on mast cells, basophils, and other inflammatory cells [22]. Mast cell and basophil granule secretion of preformed mediators such as histamine, and release from the same cells of newly generated lipid-derived mediators, such as prostaglandins and leukotrienes, stimulate the biosynthesis of vasodilator nitric oxide (NO), leading to hypotension that may progress to shock [25,29]. NO is produced by NO synthase (NOS) from arginine and directly activates vascular smooth muscle cell (VSMC) guanylyl cyclase, leading to cyclic guanosine monophosphate-induced vasorelaxation [30].

Hydrogen sulfide (H_2_S) formed from L-cysteine by either cystathionine β-synthase or cystathionine γ-lyase (CSE) [31] activates the ATP-sensitive K+ channel (KATP) of VSMC, inducing smooth muscle cells (SMC) hyperpolarization [32]. NO and H_2_S synergize in modulation of vascular tone [33]. H_2_S activates endothelial NOS (eNOS) to increase NO bioavailability [34]. NO donors can increase CSE expression to promote H_2_S biosynthesis [35].

AS-associated vasodilation secondary to allergen-induced mediator release reflects, at least in part, activation of vascular K+ channels in settings of anoxia, metabolic inhibition, and ischemia [36,37]. VSMC express several types of K+ channels, including Kv, Ca^2+^-activated K+, KATP, inward rectifier K+, and two pore domain K+ channels [38,39,40]. We previously demonstrated attenuated hypotension and improved survival in a rat model of AS treated post-challenge with KV blocker, 4-AP [37]. As H_2_S is also an important vascular tone regulator in cardiovascular and inflammatory disease [41,42], we investigated the roles of inhibitors of CSE and of NOS, alone or in combination with 4-AP, in the treatment of the lethal hypotension induced by AS in ovalbumin-sensitized Wistar rats.

## 2. Materials and Methods

### 2.1. Anaphylactic Shock Model

Experiments were performed using 4-week-old male Wistar rats (250 ± 15 g) housed in groups of four in polypropylene cages with a 12-h light-dark cycle at 24–26 °C and ad libitum food and water. After one-week’s acclimatization, rats were divided into experimental groups of six rats each. Ovalbumin (OVA, 1 mg) was dissolved in a suspension of aluminum hydroxide (Al OH; 3.5 mg) in 1 mL 0.9% sterile normal saline. The OVA-Al OH suspension (1 mL) was injected subcutaneously at subscapular sites on days 0, 5 and 14 to sensitize rats. Naive rats were injected with 1 mL suspensions of Al OH alone (36, 37). All animal experiments were conducted in accordance with protocols approved by the Institutional Review Board and the Institutional Animal Care and Research Advisory Committee of the United Arab Emirates University College of Medicine and Health Sciences, Al Ain, Abu Dhabi, United Arab Emirates.

One week after the final OVA immunization, 7-week-old rats were anesthetized with pentobarbital sodium (62.5 mg/kg) administered intraperitoneally (i.p.). Tracheas were surgically cannulated for artificial ventilation with 100% O_2_ through an endotracheal tube attached to a constant-volume ventilator (Harvard Apparatus, Edenbridge, UK) set at 60 breaths/min, with tidal volume of 6 mL/kg and expiratory pressure of 5 cm H_2_O. Body temperature was maintained at 37 °C by a thermal blanket (Harvard Apparatus, Holliston, MA, USA).

One catheter (PE10 tubing) was placed in the left jugular vein and connected to a slow injection/infusion pump (Harvard Apparatus, MA, USA) for i.v. treatment. Another catheter was placed in the left carotid artery and connected via pressure transducer to the blood pressure module of the PowerLab^®^system (AD Instruments, Bella Vista, NSW, Australia) to measure systolic, diastolic, mean arterial blood pressure (MAP), and heart rate (HR). Normal saline (0.9%) was infused i.v. at 2 mL/h by infusion pump to compensate for estimated intra-surgical fluid loss.

The surgical procedures were followed by a thirty-minute stabilization period, during which MAP and HR were measured at 5 min intervals. Following the 1 mg-OVA challenge, i.v. treatments were administered after 1 min, and hemodynamic parameters were recorded at 1-min intervals during a 60-min period.

### 2.2. Treatment Groups

Drugs were studied in sensitized rats after AS induction by i.v. bolus injection of OVA. The doses of each drug were decided from published data (63, 66, 37, 77). All drugs were administered bolus i.v. followed by i.v. pump infusion till the end of the experiment to keep the same procedure for each rat groups. The bolus i.v. injection started one minute after AS induction and was complete by t = 2 min. Experimental groups included: NA, untreated, non-sensitized rats (*n* = 6); Controls, sensitized rats treated with normal saline as an i.v. bolus, 1 min after AS induction by OVA challenge followed by saline continuous infusion (*n* = 10); 4-AP, sensitized rats treated with 4-AP (1 mg/kg, i.v. bolus followed by 1 mg/kg continuous i.v. infusion) 1 min after AS induction (*n* = 6); EPI, sensitized rats treated with epinephrine (i.v. bolus, 10 µg/kg at 1 min followed by 10 µg/kg continuous i.v. infusion)) (*n* = 10); DPG, sensitized allergic rats treated with dl-propargylglycine (50 µg/kg i.v. bolus followed by 50 µg/kg continuous i.v. infusion) (*n* = 10); BCA, allergic rats treated with ß-cyanoalanine (50 µg/kg i.v. bolus followed by 50 µg/kg continuous i.v. infusion) (*n* = 10); L-NAME, N(G)-Nitro-L-arginine methyl ester (a non-specific inhibitor of NOS, 100 mg/Kg i.v. bolus followed by 100 mg/kg continuous i.v. infusion) (*n* = 10), groups also included those treated with the following two drugs regimens: 4-AP+DPG (*n* = 10); 4-AP+BCA (*n* = 10); 4-AP+L-NAME (*n* = 10); and 4-AP+EPI (*n* = 10) with the same doses in combination treatment as a bolus followed by infusion in the same doses as described above for single-drug treatments. All groups had continuous post-surgery i.v. infusion at 2 mL/h i.v. with either drug solutions or saline as appropriate.

Whole blood samples were withdrawn from the carotid artery and immediately transferred to heparinized tubes when MAP fell below 25 mm Hg, defined as the end of the experiment, and corresponding to shock; or no later than t = 60 min for surviving rats that maintained MAP > 25 mm Hg throughout the hour. ~200 μL blood was used immediately to determine blood gas and electrolyte parameters (pH, HCO3, paO2, paCO2, K^+^), and the remaining blood was centrifuged at 3000× *g* for 15 min at 4C to separate plasma, which was stored at −80 °C until used for enzyme-linked immunosorbent assays (ELISA).

### 2.3. Plasma Mediator Concentrations

Plasma concentrations of histamine, leukotriene B4 (LTB4), prostaglandin E2 (PGE2), prostaglandin F2α (PGF2), angiotensin II, vasopressin, catalase, thiobarbituric acid-reactive substances (TBARS), 8-isoprostane, and superoxidase dismutase were measured by ELISA (Cayman Chemical, Ann Arbor, MI, USA).

### 2.4. Statistical Analysis

Results are mean ± S.E.M. statistical significance was assessed by two-way analysis of variance (ANOVA) (e.g., naive × treatment; time as a repeated measure when needed) and Bonferroni-corrected comparisons of MAP and HR experiments. The integrated MAP (area under the curve, MAP-AUC) was calculated. Normally distributed data were compared among groups by ANOVA test with Tukey HSD correction. For other data, statistical significance (*p* < 0.05) was assessed by Kruskal–Wallis–Dunn post-hoc comparison test with Bonferroni correction (SPSS, Chicago, IL, USA). Group survival portrayed by Kaplan–Meier curves was compared by Log-Rank test.

## 3. Results

Anaphylactic shock-induced severe hypotension

**Figure 1:**
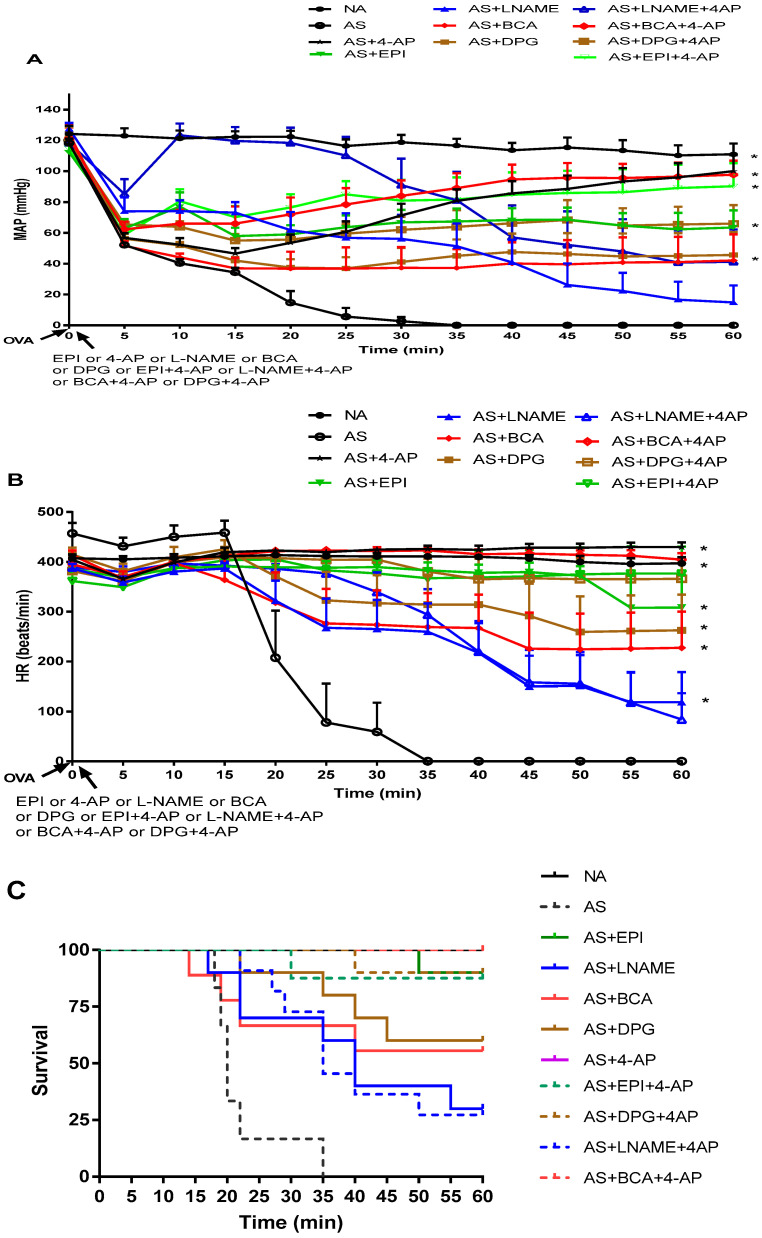
Time-dependent effects on (**A**) mean arterial blood pressure (MAP, mm Hg), (**B**) heart rate (HR, beats/min) and (**C**) survival (%) in NA (filled circles, •) or AS rats treated without (open circles, o) or with (**A**,**B**) β-cyanoalanine (BCA, filled diamonds, ♦),dl-propargylglycine (DPG, filled squares, 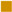), L-NG-Nitroarginine methyl ester (L-NAME, filled triangles, 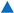), with 4-aminopyridine (4-AP, ★), epinephrine (EPI, 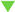) or combination drug treatment BCA+4AP (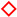), DPG+4AP (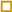), L-NAME+4AP (**Δ**), EPI+4-AP (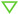) administered 1 min after induction of AS in sensitized rats by IV ovalbumin (OVA). MAP and HR were recorded every minute for 60 min post-OVA injection. Values are Mean ± S.E.M. The difference between NA and AS rats was significant (*p* < 0.0001, two-way analysis of variance, ANOVA) (**A**,**B**). The difference observed between AS and all treated groups except AS+ LNAME was significant (*p* < 0.0001) (**A**). The differences between AS and single drug treatments (4-AP, BCA, DPG, EPI) and two-drug treatments (BCA+4-AP, DPG+4-AP, EPI+4-AP) were significant (*p* < 0.0001) with L-NAME (*p* < 0.01) (**B**,**C**) Survival for each group, NA 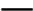, AS 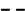, AS+EPI 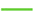, AS+LNAME 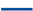, AS+BCA 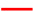, AS+DPG 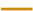, AS+4AP 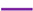, AS+ EPI+4AP 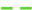, AS+DPG+4AP 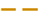, AS+LNAME+4AP 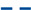, AS+BCA+4AP 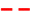. NA, non-allergic rats; AS, allergic rats; BCA, β-cyanoalanine; DPG, dl-propargylglycine; L-NAME, L-NG-Nitroarginine methyl ester; 4-AP, 4-aminopyridine; EPI, epinephrine. BCA+4-AP, β-cyanoalanine+4-aminopyridine; DPG+4-AP, dl-propargylglycine+4-aminopyridine; L-NAME+4-AP, L-NG-Nitroarginine methyl ester+4-aminopyridine; EPI+4-AP, epinephrine+4-aminopyridine.

NA group MAP (111 ± 7 mmHg) and HR (396 ± 12/min) remained unchanged throughout the 60-min experiment (Figure 1A,B), whereas the AS group responded to OVA challenge. AS rats survived 22.3 ± 3.0 min post-OVA injection; none remained alive by 35 min, whereas all NA rats survived 60 min (Figure 1C). 

### 3.1. Inhibitors of NO Biosynthesis, H_2_S Biosynthesis, and Kv Channels Attenuated Systemic Hypotension and Increased Survival in AS

After MAP decreased for 2–5 min post-OVA injection (*p* ˂ 0.001 vs. NA group) (Figure 1A), MAP stabilized in the AS-L-NAME group at ~75 mmHg for 15 min, then fell progressively to 15 ± 38 mmHg at 60 min (*p* ˂ 0.001 vs. NA group) (Figure 1A). In AS-BCA and AS-DPG groups, MAP decreased post-OVA injection~54% at 5 min and ~70% at 15 min of the baseline but stabilized during the remaining 45 min at 55 mmHg for AS-BCA and 64 ± 35 mmHg for AS-DPG (Figure 1A). MAP at 60 min was higher in the AS-4AP group (100 ± 31 mmHg) than in the AS-EPI group (64 ± 35 mmHg) (Figure 1A) and in groups treated with inhibitors of NO and H_2_S biosynthesis.

HR in all groups fell within 1 min post-OVA injection, then stabilized by 10–15 min post-injection before again decreasing (Figure 1B). The AS-EPI group maintained a normal HR until dropping slightly at 55 min (Figure 1B). AS-L-NAME group HR decreased slowly from 387 ± 29/min to 259 ± 109/min (*p* < 0.01 vs. NA), reaching118 ± 191/min at 60 min (*p* < 0.06 vs. NA; *p* < 0.02 vs. AS-EPI) (Figure 1B). BCA group HR decreased post-injection to stabilize by 25 min at 276 ± 209/min (227 ± 219/min at 60 min). AS-DPG group HR fell over 25 min until stabilizing at 323 ± 180/min (263 ± 228/min at 60 min) (Figure 1B), values similar to those of the AS-EPI group (Figure 1B). AS-4AP group HR declined only briefly post-injection before regaining initial values (*p* ˂ 0.0001 vs. NA group). AS-4-AP group HR was indistinguishable from NA group HR and remained normal for a longer period than did AS-EPI group (Figure 1B). Further, 4-AP more effectively maintained HR than did BCA or DPG.

Nine of ten EPI rats survived 60 min (mean survival 59 ± 1 min; *p* ˂ 0.0001 vs. untreated AS group) (Figure 1C). Only 30% of AS-L-NAME rats survived 60 min. AS treatment with H_2_S biosynthesis inhibitors allowed 56% of AS-BCA rats and 60% of AS-DPG rats to survive 60 min (both *p* < 0.01 vs. untreated AS group). All AS-4AP rats survived 60 min (*p* ˂ 0.0001 vs. untreated AS group) (Figure 1C).

Thus, 4-AP restored blood pressure and HR, and improved survival more effectively in our AS model than did either BCA or DPG.

### 3.2. NO and H_2_S Inhibitors Enhance Early Protective Effects of 4-AP in Anaphylactic Shock

To evaluate possible interaction of NO and H_2_S mediators with KV channels in AS-induced hypotension, we tested inhibitors of these pathways in combination. The addition of 4-AP to either L-NAME, DPG, BCA, or EPI treatments of AS modified time-dependent MAP vs. individual treatments (Figure 1A). In all groups, MAP fell within 1 min post-OVA challenge (*p* ˂ 0.04 for any AS group vs. NA group). Within 5 min post-OVA injection, MAP fell ~40% from the baseline value. In the AS-L-NAME+4AP group, MAP returned to pre-injection level within 10 min, but declined to values lower than in the NA group at 40 min (*p* ˂ 0.01) and 60 min (*p* ˂ 0.03). EPI+4AP maintained higher MAP at 60 min (90 ± 43 mmHg) than EPI alone (64 ± 35 mmHg) and differed from the untreated AS group (*p* < 0.004) (Figure 1A). Similarly, AS-BCA+4-AP preserved MAP at 60 min (~100 ± 28 mmHg) better than BCA alone (46 ± 47 mmHg; Figure 1A). DPG+4-AP preserved MAP at 60 min (67 ± 30 mmHg) better than DPG alone (46 ± 47 mmHg; Figure 1A), as L-NAME+4AP also improved MAP at 60 min (41 ± 66 mmHg) better than L-NAME alone (22 ± 38 mmHg). The addition of 4AP to EPI and to BCA similarly improved MAP (Figure 1A).

HR fell in all treated groups within 1-min post-injection but normalized by 10 min with addition of 4AP to EPI, DPG and BCA groups (Figure 1B). Further, 4-AP addition improved 60 min survival (Figure 1B) from 60% to 90% for DPG, and 56% to 100% for BCA. 60 min survival of AS-L-NAME rats was also improved from 30% to 50% by 4-AP addition. Meanwhile, 4AP combined with H_2_S inhibitors was more effective in restoring HR than H_2_S inhibitors alone (AS+4-AP vs. AS+4-AP+BCA *p* ˂ 0.0001, AS+4-AP vs. AS+4-AP+DPG, *p* ˂ 0.0001).

### 3.3. Combined 4-AP Treatments Increase Time-Integrated MAP (AUC-MAP)

**Figure 2:**
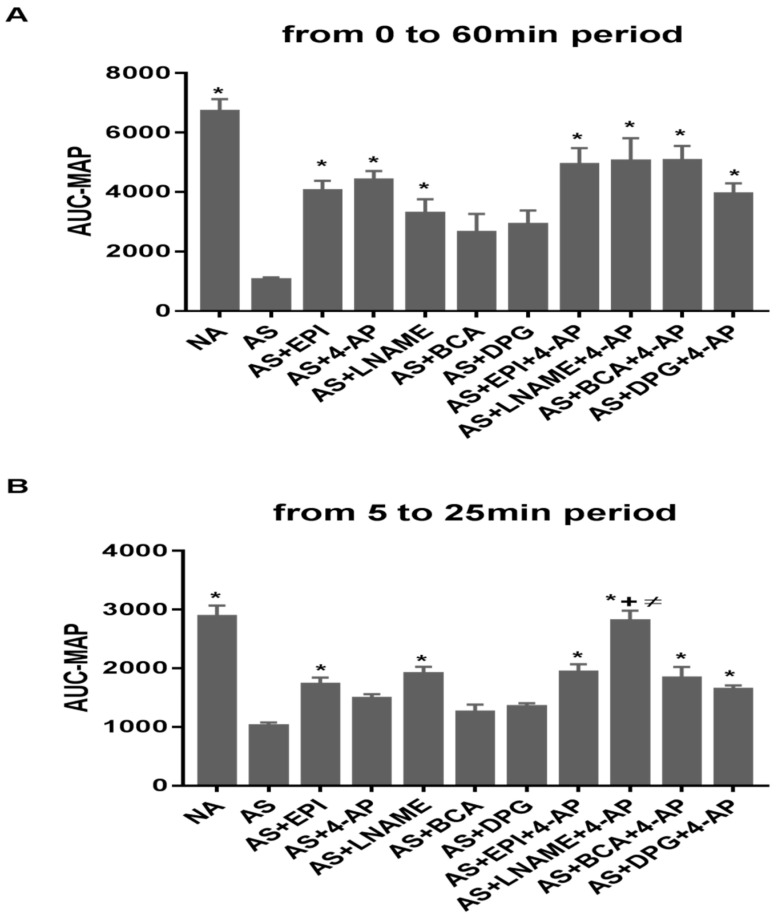

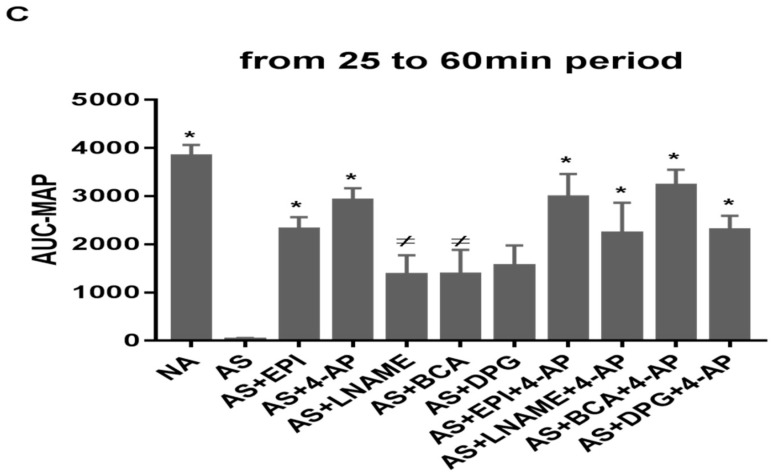
Integrated mean arterial blood pressure (area under the curve, AUC-MAP) during the full 60 min experiment (**A**), during the early experimental period from 5 to 25 min, (**B**) or during the late experimental period from 25 to 60 min (**C**). * *p* < 0.05; significantly different vs. AS group; +, *p* < 0.05 vs. EPI group; ≠, *p* < 0.05 vs. 4-AP group.

AUC-MAP was calculated over 60 min (Figure 2A), from 5 to 25 min (Figure 2B) and from 25 to 60 min (Figure 2C). AUC-MAP for the entire 60 min experimental period (Figure 2A) was lower in AS than NA groups (*p* ˂ 0.0001). AUC-MAP of the EPI, 4-AP, and L-NAME groups was higher than for AS group (*p* ˂ 0.002, *p* ˂ 0.0004, *p* ˂ 0.04 respectively). BCA and DPG treatments did not affect AUC-MAP. Combined treatments showed higher AUC-MAP than for AS group (*p* ˂ 0.0001 for AS vs. AS+EPI+4-AP, vs. AS+L-NAME+4-AP, vs. AS+BCA+4-AP; *p* ˂ 0.003 vs. AS+DPG+4-AP).

Between 5 and 25 min, AUC-MAP (Figure 2B) was lower in AS than in NA groups (*p* ˂ 0.0001). AUC-MAP was higher in EPI and L-NAME groups than controls (*p* ˂ 0.009 and *p* ˂ 0.0006, respectively). BCA, DPG, and 4-AP treatments did not affect AUC-MAP. All 4-AP combination treatments showed higher AUC MAP values than for AS group (*p* ˂ 0.0007 vs. AS+EPI+4-AP, *p* ˂ 0.0001 vs. AS+L-NAME+4-AP, *p* ˂ 0.001 vs. AS+BCA+4-AP, *p* ˂ 0.03 vs. AS+DPG+4-AP).

Between 25 min and 60 min (Figure 2C), AUC-MAP was lower in AS than in NA groups (*p* ˂ 0.0001). EPI and 4-AP treatments increased AUC-MAP vs. AS group (*p* ˂ 0.003 and *p* ˂ 0.0001, respectively). L-NAME, BCA, and DPG did not affect AUC-MAP. Combined 4-AP treatment AUC-MAP values exceeded those of AS group (*p* ˂ 0.0002 vs. AS+EPI+4-AP, *p* ˂ 0.005 vs. AS+L-NAME+4-AP, *p* ˂ 0.0001 vs. AS+BCA+4-AP *p* ˂ 0.003 vs. AS+DPG+4-AP).

### 3.4. Metabolic Acidosis Was Not Rescued by Treatment

**Figure 3:**
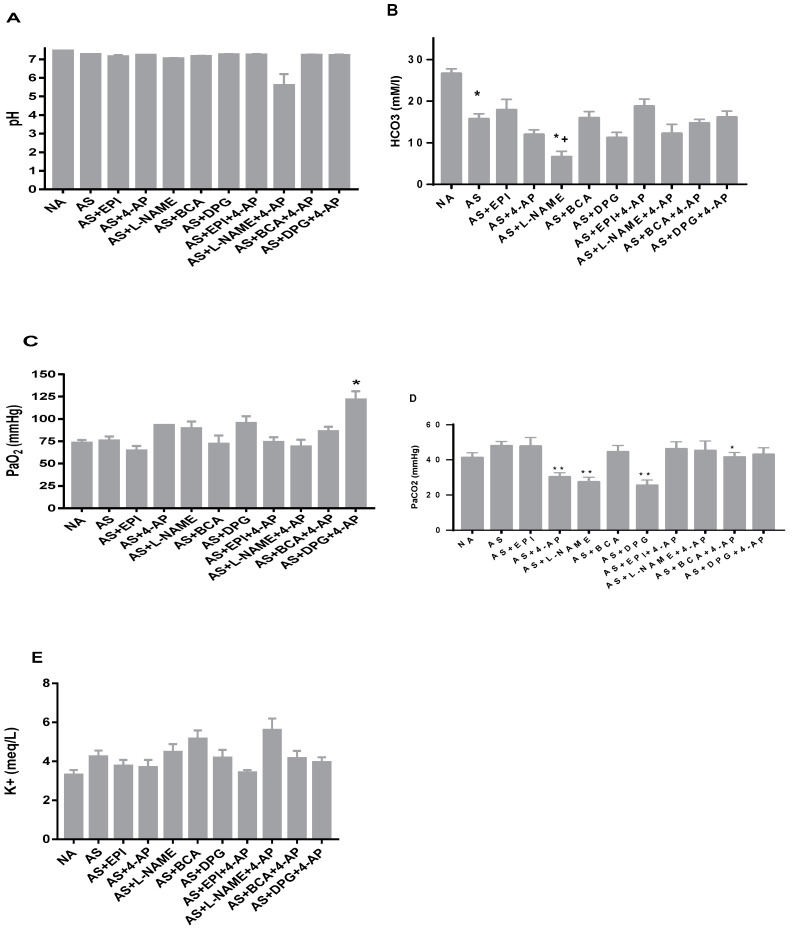
Arterial blood levels of pH (**A**), [HCO3–] (**B**), pO2 (**C**), pCO2 (**D**) and [K+] (**E**) after injection of ovalbumin in non-allergic (NA), allergic rats (AS), and rats treated with L-NAME, DPG, BCA, 4-AP or EPI, and rats treated with the 4-AP drug combinations L-NAME+4-AP, DPG+4-AP, BCA+4-AP, or EPI+4-AP. Values are Mean ± S.E.M; * *p* < 0.05 vs. AS group, ** *p* < 0,001 vs. AS group, + *p* < 0.05 vs. EPI group, ≠ *p* < 0.05 vs. 4-AP group. NA, non-allergic rats; AS, allergic rats; BCA, β-cyanoalanine; DPG, dl-propargylglycine; L-NAME, L-NG-Nitroarginine methyl ester; 4-AP, 4-aminopyridine; EPI, epinephrine. BCA+4-AP, β-cyanoalanine+4-aminopyridine; DPG+4-AP, dl-propargylglycine+4-aminopyridine; L-NAME+4-AP, L-NG-Nitroarginine methyl ester+4-aminopyridine; EPI+4-AP, epinephrine+4-aminopyridine.

The AS group exhibited severe metabolic acidosis ([HCO3–] 15.7 ± 1.24 mM and pH 7.2 ± 0.03), reflecting AS-associated tissue hypoperfusion (Figure 3A,B). AS-induced metabolic acidosis was not rescued by any tested treatment (Figure 3A). However, L-NAME further exacerbated metabolic acidosis (Figure 3A) and lowered serum [HCO3-] (*p* ˂ 0.01 vs. untreated AS) (Figure 3B). PaO2 was elevated by combined treatment with DPG+4-AP but was unchanged with other treatments (Figure 3C). PaCO2 was lower in rats treated with LNAME, DPG and 4-AP as compared to untreated rats (Figure 3E, *p* < 0.001). Serum K+ was unchanged by any treatments (Figure 3D).

### 3.5. NO, H_2_S and Kv Inhibitors Differentially Alter Oxidative Stress

**Figure 4:**
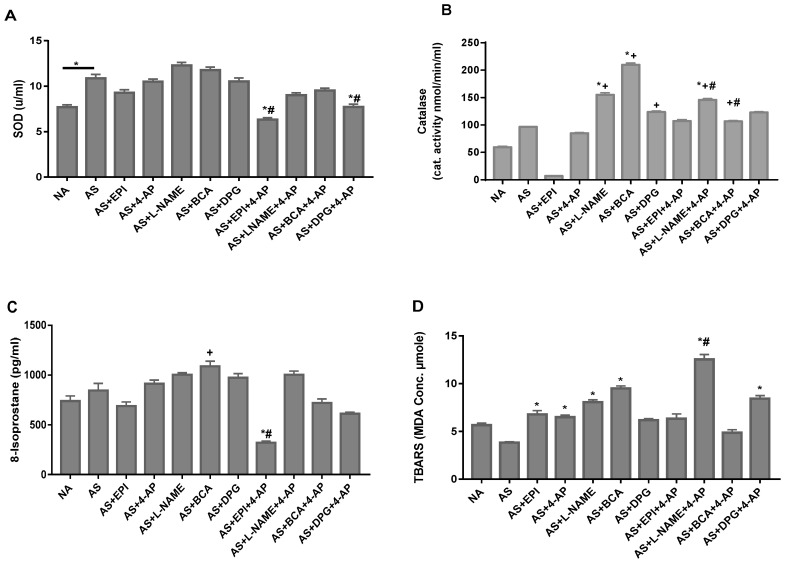
NA and AS rat arterial blood levels of the oxidative stress markers, superoxide dismutase (SOD) (**A**), catalase (**B**), 8-isoprostane (**C**), and thiobarbituric acid-reactive substances (TBARS) (**D**) after OVA injection in non-allergic (NA) and allergic rats (AS), followed 1 min afterwards with injection of EPI, L-NAME, BCA, DPG, or 4-AP, or (in rats treated with 4-AP drug combinations) L-NAME+4-AP, BCA+4-AP, DPG+4-AP or EPI+4-AP. Values are Mean ± S.E.M; * *p* < 0.05 vs. AS group, + *p* < 0.05 vs. EPI group, # *p* < 0.05 vs. 4-AP group. NA, non-allergic rats; AS, allergic rats; BCA, β-cyanoalanine; DPG, dl-propargylglycine; L-NAME, L-NG-Nitroarginine methyl ester; 4-AP, 4-aminopyridine; EPI, epinephrine. BCA+4-AP, β-cyanoalanine+4-aminopyridine; DPG+4-AP, dl-propargylglycine+4-aminopyridine; L-NAME+4-AP, L-NG-Nitroarginine methyl ester+4-aminopyridine; EPI+4-AP, epinephrine+4-aminopyridine.

To assess oxidative stress caused by reactive oxygen species (ROS), we measured plasma levels of ROS, 8-isoprostane, TBARS and the antioxidant enzymes, catalase and superoxide dismutase (SOD). SOD plasma levels were significantly increased in untreated AS rats (Figure 4A). Neither BCA nor DPG produced any effect on plasma SOD, whereas the treatment of AS rats with 4-AP+DPG or 4-AP+EPI (Figure 4A) unexpectedly decreased SOD. Although plasma levels of catalase were normal in untreated rats, we observed increased catalase levels in rats treated with BCA, DPG, L-NAME, and with 4-AP + LNAME (Figure 4B). Plasma levels of ROS, TBARS were increased in 4-AP, L-NAME, BCA, EPI, and 4-AP+DPG rats (Figure 4C,D). Thus, we found an effect of ICSE, L-NAME, or 4-AP on plasma levels of TBARS markers and catalase.

### 3.6. Inhibitors of NO, H_2_S Biosynthesis, or Kv Channels Differentially Alter Plasma Inflammatory Mediators

**Figure 5:**
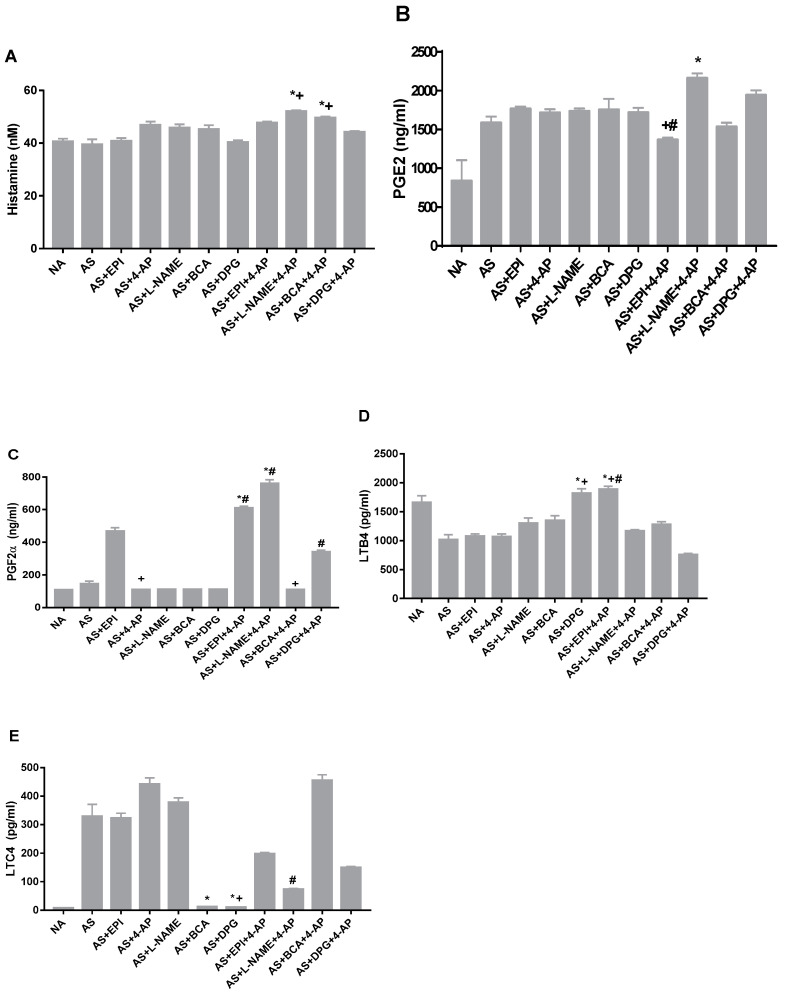
AS arterial plasma concentrations of the allergic and inflammatory mediators histamine (**A**), prostaglandin E2 (PGE2) (**B**), prostaglandin F2α (PGF2α) (**C**), leukotriene B4 (LTB4) (**D**), and leukotriene C4 (LTC4) (**E**) after OVA injection in untreated AS rats, rats treated with EPI, L-NAME, DPG, BCA,4-AP or EPI, and rats treated with the 4-AP drug combinations with L-NAME+4-AP, BCA+4-AP, DPG+4-AP or EPI+4-AP. Values are Mean ± S.E.M; * *p* < 0.05 vs. AS group; + *p* < 0.05 vs. EPI group; # *p*< 0.05 vs. 4-AP group. NA, non-allergic rats; AS, allergic rats; BCA, β-cyanoalanine; DPG, dl-propargylglycine; L-NAME, L-NG-Nitroarginine methyl ester; 4-AP, 4-aminopyridine; EPI, epinephrine. BCA+4-AP, β-cyanoalanine+4-aminopyridine; DPG+4-AP, dl-propargylglycine+4-aminopyridine; L-NAME+4-AP, L-NG-Nitroarginine methyl ester+4-aminopyridine; EPI+4-AP, epinephrine+4-aminopyridine.

No individual or combined treatments altered histamine levels during AS (Figure 5A). Whereas no individual treatments altered PGE2 levels (Figure 5B), PGE2 levels increased in group LNAME+4-AP. PGF2α plasma levels during AS were unaltered by individual treatments (Figure 5C) and markedly elevated by LNAME+4-AP (*p* ˂ 0.0007) or EPI+4AP (*p* ˂ 0.01 vs. AS).

LTB4 levels were elevated by AS treatment with DPG (*p* < 0.0002, Figure 5D), but this elevation was suppressed by combined DPG+4-AP. AS treatment with EPI+4-AP also increased LTB4 (*p* ˂ 0.0001; Figure 5D). In contrast, Leukotriene C4 (LTC4) levels were nearly undetectable in AS rats treated with DPG or BCA (*p* < 0.0003; Figure 5E), effects attenuated by added 4-AP. These observations may indicate direct or indirect interactions between H_2_S and lipoxygenase pathway components in AS.

### 3.7. AP Increases Angiotensin II and Vasopressin Plasma Levels in the Rat AS Model

**Figure 6:**
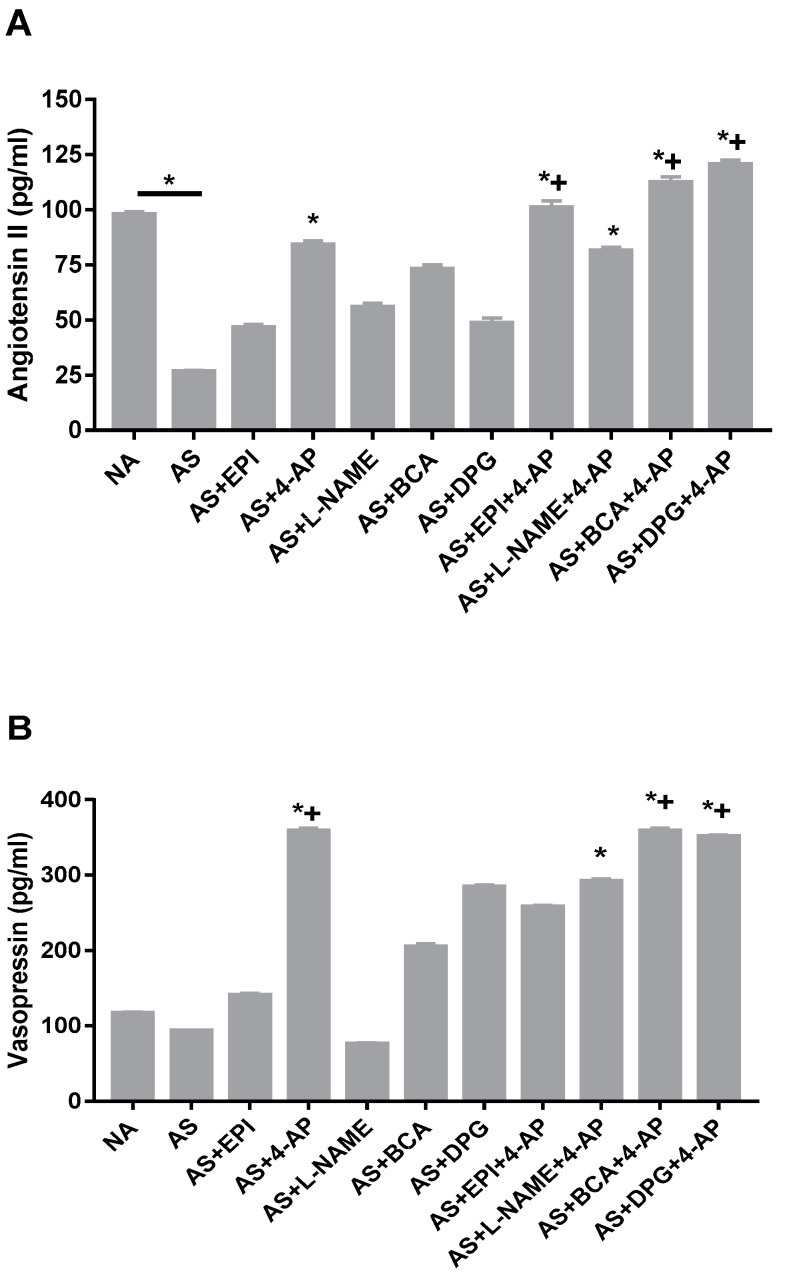
Arterial plasma concentrations of angiotensin II (**A**) and vasopressin (**B**) after OVA injection in non-allergic (NA) or allergic rats (AS) treated with EPI, L-NAME, DPG, BCA, 4-AP or EPI, and in rats treated with the 4-AP drug combinations L-NAME+4-AP, DPG+4-AP, BCA+4-AP, or EPI+4-AP. Values are Mean ± S.E.M; * *p* < 0.05 vs. AS group; + *p* < 0.05 vs. EPI group; ≠ *p* < 0.05 vs. 4-AP group. NA, non-allergic rats; AS, allergic rats; BCA, β-cyanoalanine; DPG, dl-propargylglycine; L-NAME, L-NG-Nitroarginine methyl ester; 4-AP, 4-aminopyridine; EPI, epinephrine. BCA+4-AP, β-cyanoalanine+4-aminopyridine; DPG+4-AP, dl-propargylglycine+4-aminopyridine; L-NAME+4-AP, L-NG-Nitroarginine methyl ester+4-aminopyridine; EPI+4-AP, epinephrine+4-aminopyridine.

AS decreased angiotensin II plasma levels (*p* < 0.0001 vs. NA), whereas vasopressin levels remained unchanged (Figure 6A,B). Further, 4-AP treatment of AS increased the plasma level of angiotensin II (Figure 6A; *p* < 0.001) and vasopressin (Figure 6B; *p* < 0.0001), and both elevated levels were sustained during combined treatment with 4-AP and any other single agent. These data suggest that Kv inhibition may help restore systemic blood pressure by elevating vasoconstrictors blood level to protect against AS-induced hypotension.

## 4. Discussion

Our experiments confirm our previous findings demonstrating that Kv channel inhibition with 4-AP improves hypotension and survival in the Wistar OVA rat model of anaphylactic shock (AS). In contrast, single agent administration of ICSE, BCA, or DPG is of lower efficacy than 4-AP in the treatment of AS. The greater efficacy of BCA as compared to DPG supports the finding of Asmakopoulo et al. that BCA inhibits CSE (IC50 14 ± 0.2 μM) more potently than DPG (40 ± 8 μM) [43]. The combination of 4-AP with ICSE BCA or EPI sustained anti-hypotensive and survival benefits of 4-AP at 60 min post-challenge, while further attenuating the maximal decline in blood pressure during single agent 4-AP treatment at 15–30 min post-challenge. The enhanced effects of combination treatment of AS on post-challenge survival and hypotension may reflect the accompanying elevations in serum levels of the vasoconstrictors angiotensin II and vasopressin. The increase in PGF2α observed in rats treated with 4-AP+EPI or 4-AP+L-NAME and the increase in LTB4 in rats treated with 4-AP+EPI may also contribute to improved outcomes.

Endothelial cells of the vascular intima control vascular tone through actions of endothelial mediators (e.g., nitric oxide, prostacyclin), vasoactive metabolites (acidosis, hypoxia, hydrogen peroxide), autacoids (histamine, serotonin, prostaglandins, thromboxane A2, leukotrienes), and sympathetic nerves, along with vasoactive hormonal mediators such as epinephrine, angiotensin II, and vasopressin [44]. Reductions in cytosolic [Ca^2+^] induce vasodilation whereas elevated cytosolic [Ca^2+^] leads to vasoconstriction. Relaxation of vascular smooth muscle cells (VSMC) is associated with efflux Ca^2+^ through Plasma Membrane Ca^2+^ ATPase-4 Ca^2+^-ATPases and sodium-calcium exchanger, NCX1, as well as by organellar Ca^2+^ sequestration by SERCA and perhaps other Ca^2+^ ATPases [45,46,47,48].

AS mediators released by mast cells and basophils activate receptors on endothelial cells and VSMC, inducing vasodilation with increased capillary permeability, leading to severe hypotension and hypovolemia [45]. The gold standard of AS treatment is epinephrine, a vasopressor interacting with α- and β-adrenergic receptors. Activation of α1-adrenoceptors located on VSMC increases blood pressure by inducing vasoconstriction [49,50,51]. In our rat AS model, EPI alone or in association with 4-AP treatment restores blood pressure and improves survival through α1-adrenoceptor stimulation and Kv channel inhibition.

Vasodilation induced by the mediators released by IgE-activated mast cells and basophils is associated with increased intracellular cAMP, activating protein kinase A (PKA). PKA opens the Kv channels to hyperpolarize the plasma membrane, inactivating CaV1.2 voltage-gated Ca^2+^ channels and thereby decreasing intracellular [Ca^2+^] [49]. PKA phosphorylates MLCK and decreases its calmodulin affinity [49]. Prostacyclin (PGI2, not measured in our experiments) and PGE2, through the respective actions of cyclo-oxygenase (COX) 2 and COX1, respectively, are released during degranulation of mast cells and basophils. PGI2 and PGE2 are also produced by endothelial cells and VSMC via plasma membrane arachidonic acid metabolism, activating guanylate cyclase to generate cGMP, which can activate nitric oxide synthase (NOS) to generate NO, as regulated by intracellular [Ca^2+^] [52,53,54]. Endothelial cell NO diffuses to VSMCs and vasodilates blood vessels through activation of cGMP-dependent PKGII [55,56,57,58]. eNOS can also be activated by PKA-mediated phosphorylation [59,60].

Systemic infusion of L-NAME can increase blood pressure [61,62] through NOS inhibition and decreased NO production. In our experiments, however, L-NAME failed to normalize blood pressure and survival at 60 min. 4-AP combined with L-NAME showed modest efficacy in defending blood pressure during the early period between 5–25 min, but this effect subsequently subsided during the later period out to 60 min. L-NAME increased AUC-MAP during early phase AS, but 4-AP sustained increased AUC-MAP throughout the experimental period. Although combined treatments increased AUC-MAP throughout, L-NAME+4-AP was more effective during the first 25 min. L-NAME alteration of blood pressure and vascular reactivity likely reflects generalized NOS inhibition. Only constitutive eNOS and nNOS forms were likely activated in our AS model, since the 1 h experimental period may have been too brief for iNOS activation [63,64]. L-NAME improves hemodynamics at early time points but does not increase survival. L-NAME attenuated reduced arterial [HCO3-] and increased catalase levels without effect on ROS and inflammatory mediators. These effects appear less important than reported previously in other animal models of AS [63,64,65,66]. They also contrast with the reported detrimental effects of L-NAME in non-AS rats [67] and with the deleterious effects of NO in guinea pig AS [68]. NOS activation kinetics may explain why L-NAME efficacy was restricted to earlier times. AS rats exhibit infiltration of mast cells and eosinophils in lung, small bowel mucosa and spleen, with less infiltration in heart tissue. These pathological findings were associated with the increased expression of eNOS and iNOS in vascular endothelial cells, mostly in lung, heart and small bowel wall [69].

Arachidonic acid is a major cell membrane component released by phospholipase A2 enzyme [70]. The metabolism of arachidonic acid produces prostaglandins/thromboxanes, leukotrienes, and other mediators, such as 20-hydroxyeicosatetraenoic acid, epoxyeicosatrienoic acid, and isoprostanes. PGF2α, LTB4, and LTC4 induce vasoconstriction, whereas PGE2 and prostacycline (PGI2) lead to vasodilation. In our model, PGE2 was not altered by treatment with 4-AP, L-NAME, DPG, BCA, or EPI. In contrast, PGE2 was increased in AS rats treated with 4-AP+L-NAME, whereas the combinations of 4-AP+EPI, 4-AP+DPG, or 4-AP+BCA were without effect. The vasoconstrictor PGF2α was increased in AS rats treated with 4-AP+EPI or with 4-AP+L-NAME. The combination of 4-AP with EPI was associated with increased serum levels of vasoconstrictor leukotriene LTB4 [71,72]. AS rat treatment with 4-AP+EPI was associated with increased PGF2α and LTB4, possibly contributing to normalization of blood pressure and survival.

Inhibition of H_2_S production, alone or in combination with 4-AP, was unassociated with increases in PGF2α, LTC4, or LTB4. LTB4-mediated recruitment of inflammatory cells [72] and LTC4-induced vascular leakage and reduction in cardiac perfusion [22] can contribute to myocardial depression and ventricular dysfunction. The increased LTB4 and decreased LTC4 associated with DPG treatment of AS (Figure 5) may produce opposing effects on vascular tone. The interaction of prostaglandins and leukotrienes with H_2_S and NO in vasodilation remains unclear and requires further investigation.

Combination treatments produced larger increases in AUC-MAP during both early and late experimental periods (Figure 2), with addition of the ICSE BCA increasing AUC-MAP. Our results suggest that Kv inhibitor treatment combined with BCA may be more effective than individual ICSE treatments. H_2_S can directly activates KATP channels in some VSMC [29,54] to promote membrane hyperpolarization and inactivation of CaV1.2 channels to relax VSMCs [73]. H_2_S-induced cAMP elevation also relaxes smooth muscle via PKA activation [74] and activates Kv channels to vasodilate coronary arteries in rat [75] and pig [76]. DPG and BCA attenuated hypotension and improved recovery from hemorrhagic shock in Sprague-Dawley rats [77]. H_2_S can be either anti- or pro-oxidant [78,79]. At low concentrations, H_2_S exerts protective cardiovascular effects but can be cytotoxic at higher concentrations [78]. Thus, H_2_S is a SOD substrate, binding at its catalytic Cu^2+^ site [80]. H_2_S can thus delay accumulation of lipid peroxidation products [81], including TBARS, in hemin-mediated oxidation of HUVEC cells [82], and may inhibit p66-Shc-dependent mitochondrial ROS production [80,82]. H_2_S addition also increased catalase activity in rat myocardium and human endothelial cells [83], whereas BCA and DPG increased catalase in our rat AS model (Figure 4B).

Severe hypotension and bradycardia in our rat AS model led to death of all untreated allergic rats in the current and previous experiments [36,37]. We now report that our AS model is accompanied by decreased levels of the vasoconstrictor angiotensin II. The recovery of a normal blood pressure improving survival with 4-AP alone, ICSE+4-AP or 4-AP+EPI treatment could be explained by elevated plasma levels of vasoconstrictors, countering AS-induced reduction in angiotensin II and vasopressin (Figure 6). Plasma vasoconstrictors angiotensin II and vasopressin in AS were also increased by 4-AP, but not by EPI. Inhibition of angiotensin II and vasopressin action increased AS severity in rats [84], suggesting central roles for angiotensin II and vasopressin in protection against AS. The vasopressin deficiency characterizing some shock states [85,86,87] diminishes stimulation of V1 receptor-mediated protein kinase C activation and elevation of cytosolic [Ca^2+^], leading to decreased vasoconstriction [88,89]. Elevated [Ca^2+^], through the action of calmodulin, activates myosin light chain kinase (MLCK), which in turn phosphorylates myosin light chain [90], enhancing actinomyosin ATPase activity and contraction. [Ca^2+^]i elevations are critical for phospholipase A2 activation [91]. Besides activating phospholipases A2 and C, vasopressin also stimulates phospholipase D (PLD) activity in VSMC [92]. PLD contributes to long-term activation of PKC [93]. This finding suggests that in addition to directly stimulating contractile pathways, vasopressin inhibits vasodilatory pathways. Further studies are required to dissect the post-receptor pathways mediating this effect in our model. 

Angiotensin II can inhibit Kv channels via Ca^2+^-independent PKCε activation [91] and has successfully increased blood pressure in vasodilatory shock resistant to classical vasopressors [94]. Angiotensin II activates various signaling pathways through AT1R, including G-protein-derived second messengers, protein kinases and small G-proteins. Stimulation of AT1R leads to vasoconstriction via the inhibition of adenylate cyclase, decreasing vasodilator cAMP. Lipoxygenase-derived eicosanoids can contribute to the constrictor effects of Angiotensin II [95,96,97,98].

Taken together, our results suggest that an increase of PGF2α, LTB4, angiotensin II, and vasopressin could be involved in the contractile effect on VSMC, explaining the blood pressure normalization and improved survival of AS rats treated with 4-AP alone or in combination with BCA or EPI. 

Our study presents several limitations. First, our anesthetized and ventilated rat model likely experiences less hypoxia relative to the severity of hypotension than humans in severe AS prior to treatment. Second, drug administration to sensitized rats 1-min post-OVA challenge should be tested at longer times post-antigen challenge to mimic clinical presentation. Third, anesthesia masks potential epileptogenic effects of 4-AP, so we are currently testing Kv inhibitors with lower blood–brain barrier penetration. Fourth, rats exhibit higher stress-induced iNOS activation than mice and humans [99], and our L-NAME experiments failed to discriminate among NOS isoforms in this model of AS. Lastly, our experiments have not defined the exact roles of increased levels of angiotensin II and vasopressin relative to anaphylactic mediators. The release of vasodilators as PGE2, prostacyclin, and vasoconstrictor, such as PGF2α, Thromboxane A2, LTC4, LTB4, along with angiotensin II and vasopressin, should be measured at early and late time points of AS and in treated rats. Further experiments are needed to demonstrate the causal effect of this observation by using inhibitors or by reproducing AS in rats with down-regulated mediator genes.

## 5. Conclusions

We have demonstrated pharmacologically that inhibition of Kv channels combined with ICSE BCA restores blood pressure and improves survival. Release of H_2_S may contribute to the pathophysiology of AS in rat. Individual ICSE treatment with BCA or DPG is partially effective. In combination with 4-AP, BCA attenuates AS-induced cardiovascular dysfunction and allows complete cardiovascular recovery, as compared to single-agent ICSE. This protective cardiovascular effect may reflect the increase of plasma vasoconstrictors, namely angiotensin II and vasopressin. Further experiments are needed to confirm this finding and to explore the respective roles of vasodilator and vasoconstrictor prostaglandins and leukotrienes. We propose that combined treatment with 4-AP + BCA could be an alternative treatment of AS refractory to epinephrine.

## Data Availability

The data presented in this study are available on request from the corresponding author (A.B.).
